# Evolution of the CD163 family and its relationship to the bovine gamma delta T cell co-receptor WC1

**DOI:** 10.1186/1471-2148-10-181

**Published:** 2010-06-15

**Authors:** Carolyn TA Herzig, Ray W Waters, Cynthia L Baldwin, Janice C Telfer

**Affiliations:** 1 University of Massachusetts Amherst, Department of Veterinary and Animal Sciences, Paige Laboratory, Amherst, MA 01003, USA; 2 National Animal Diseases Center, USDA-ARS, Ames, Iowa, 50010, USA

## Abstract

**Background:**

The scavenger receptor cysteine rich (SRCR) domain is an ancient and conserved protein domain. CD163 and WC1 molecules are classed together as group B SRCR superfamily members, along with Spα, CD5 and CD6, all of which are expressed by immune system cells. There are three known types of CD163 molecules in mammals, CD163A (M130, coded for by *CD163*), CD163b (M160, coded for by *CD163L1*) and CD163c-α (CD163L1 or SCART), while their nearest relative, WC1, is encoded by a multigene family so far identified in the artiodactyl species of cattle, sheep, and pigs.

**Results:**

We annotated the bovine genome and identified genes coding for bovine CD163A and CD163c-α but found no evidence for CD163b. Bovine CD163A is widely expressed in immune cells, whereas CD163c-α transcripts are enriched in the WC1+ γδ T cell population. Phylogenetic analyses of the CD163 family genes and WC1 showed that CD163c-α is most closely related to WC1 and that chicken and platypus have WC1 orthologous genes, previously classified as among their CD163 genes.

**Conclusion:**

Since it has been shown that WC1 plays an important role in the regulation of γδ T cell responses in cattle, which, like chickens, have a high percentage of γδ T cells in their peripheral blood, CD163c-α may play a similar role, especially in species lacking WC1 genes. Our results suggest that gene duplications resulted in the expansion of CD163c-α-like and WC1-like molecules. This expanded repertoire was retained by species known as "γδ T cell high", but homologous SRCR molecules were maintained by all mammals.

## Background

The CD163 family includes genes encoding CD163A (also known as M130, HbSR, and coded for by *CD163*), CD163b (also known as M160, and coded for by *CD163L1*) and CD163c-α (also known as CD163L1 and SCART). This family is a subset of the scavenger receptor cysteine-rich (SRCR) super-family, an ancient super-family defined by the presence of 100-110 amino acid domains [1]. Members of the CD163 family are group B SRCR proteins distinguished by the 6-8 cysteines in their SRCR domains resulting in 3-4 disulfide bonds; in comparison, group A SRCR proteins have only 6 cysteines and 3 disulfide bonds in their SRCR domains. SRCR domains, like Ig domains or epidermal growth factor-like domains, are thought to be involved in protein-protein interactions, although the known ligands of these domains vary widely.

CD163A is a receptor for haptoglobin-hemoglobin complexes, and is inducibly expressed on monocytes, macrophages and a subpopulation of hematopoietic progenitors [2-6]. CD163A protects against oxidative damage by mediating the endocytosis of haptoglobin-hemoglobin complexes [7]. Ligation of CD163A by haptoglobin-hemoglobin complexes induces the secretion of the anti-inflammatory cytokine IL-10 [8]. In addition, proteolytically-cleaved CD163A shed into serum inhibits phorbol ester-induced T cell proliferation [9]. The third SRCR domain of CD163A mediates its interaction with haptoglobin and TWEAK (TNF-like weak inducer of apoptosis) [10,11]. CD163A interacts with a molecule expressed on erythroblasts and with bacteria via its second SRCR domain [12,13]. The related group B SRCR molecule CD6 also binds to bacteria via one or more of its SRCR domains, through interactions with the bacterial non-peptiditic products lipoteichoic acid (LTA) and lipopolysaccharide (LPS) [14].

CD163A transcripts display alternative splicing of the extracellular and cytoplasmic coding regions, potentially increasing the diversity of its function [15,16]. The CD163A form with a short cytoplasmic domain predominates and mediates ligand internalization and degradation but the function of the CD163A form with a long cytoplasmic domain is unknown [17]. Cross-linking of CD163A induces inositol triphosphate and cytokine production [18]. The CD163A cytoplasmic domain is phosphorylated by casein kinase II and protein kinase C-α (PKC-α) and these phosphorylation events are tied to cytokine production induced by CD163A cross-linking [19]. Casein kinase II activity and anti-inflammatory cytokine production in macrophages is stimulated by CD163A binding to only one of the two alleles of haptoglobin, although both bind to CD163A with high affinity [20]. The non-stimulatory haptoglobin allele is correlated with increased susceptibility to cardiovascular disease [21,22].

CD163b is also expressed on macrophages, with two cytoplasmic domain variants of 71 and 39 amino acids, resulting from alternative splicing [23]. Little is known about the function or ligand of CD163b. Thus far, the gene encoding CD163b has only been found in the genomes of primates and the horse.

Unlike CD163A, which is encoded by one gene across eutherian mammals, several species possess multiple genes encoding CD163c-α molecules. There are two CD163c-α genes in mice: SCART1 and SCART2. SCART1 is expressed in the lymph node, trachea and lung; SCART2 is expressed on murine γδ T cells that secrete IL-17 [24,25]. The presence of multiple genes encoding the SRCR transmembrane receptor CD163c-α is similar to that of another set of group B SRCR proteins, WC1. Genes encoding WC1 have been found in the artiodactyl species cattle (*Bos taurus*), sheep (*Ovis aries*) and swine (*Sus scrofa*) [26-30]. WC1 molecules are encoded by a family of fifteen genes in the bovine and, like SCART2, are expressed on γδ T cells [26,27,30-34]. WC1 contributes to the γδ T cell response to *Leptospira *(Wang F, Herzig CTA, Hsu H, Chen C, Baldwin CL, Telfer JC: Scavenger receptor WC1 contributes to the gamma delta T cell responses to *Leptospira*, submitted) and WC1-mediated potentiation of T cell activation requires the phosphorylation of a tyrosine in its cytoplasmic domain [35]. Moreover, expression of different molecular forms of WC1 on bovine γδ T cells is correlated with differential response to bacteria, suggesting that WC1 functions as a pattern recognition molecule similar to the related SRCR molecules CD163A, CD5, CD6, Spα and DMBT1 [13,14,36-40]. No WC1 homologues have thus far been identified in human or murine γδ T cells, leading to the question of whether CD163 family members, particularly CD163c-α molecules, have evolved to serve functions equivalent to WC1 in mammals other than the artiodactyls.

In our recent annotation of the bovine genome we found the gene encoding CD163A embedded within the region coding for WC1 genes [31]. To determine the extent of the CD163 family in artiodactyls, we annotated the bovine genome to identify CD163 family genes in cattle. We found genes encoding both CD163A and CD163c-α, but not CD163b, and evaluated their expression profile in γδ T cells and other tissues. Incorrect assignment of genes belonging to the CD163 and the WC1 families from many species has created substantial confusion in naming and categorizing these genes. To appropriately categorize the genes identified both by us and by others, we undertook phylogenetic analyses of CD163 and WC1 family members. Here, we show the relationship between CD163A, CD163c-α and WC1 family receptors, all expressed in γδ T cells of artiodactyls, that WC1 orthologues are present in the chicken and platypus, and correlate the conservation over evolutionary time of a diverse array of these receptors with the presence of a high level of γδ T cells in the peripheral blood.

## Results

### Exon-intron structure of bovine CD163 family members

**Figure 1:**
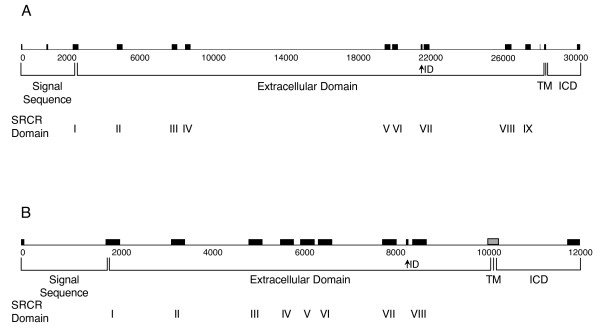
**Schematic representation of bovine CD163 exon-intron structure**. Exon-intron structures of (A) CD163A and (B) CD163c-α were determined based on annotation of the CD163 genes in the bovine genome assembly Btau_3.1. Proposed placement of the exon encoding CD163c-α transmembrane region is shown in grey and could not be confirmed due to a gap in the genomic sequence assembly. SRCR domain numbers are indicated by roman numerals. Scale is shown in base pair increments beneath the schematic. Abbreviations are as follows: ID, interdomain sequence; TM, transmembrane region; ICD, intracytoplasmic domain.

Annotation of the bovine genome indicated that cattle have a gene coding for CD163A, which was found on chromosome 5 within one of the two loci coding for the large WC1 family of genes [31]. A gene coding for bovine CD163c-α was also found but could not be placed in the bovine genome due to insufficient scaffolding. Schematics of the exon-intron structure of these two genes are shown in Fig. 1. Both CD163A and CD163c-α contain a cytoplasmic domain coded for by two exons, one of which also encodes the transmembrane domain. Interestingly, like WC1, both CD163A and CD163c-α contain an exon that encodes interdomain sequence (Fig. 1, ID) [31]. The gene encoding CD163A spans approximately 30 kbp; that of CD163c-α spans approximately 12 kbp.

**Figure 2:**
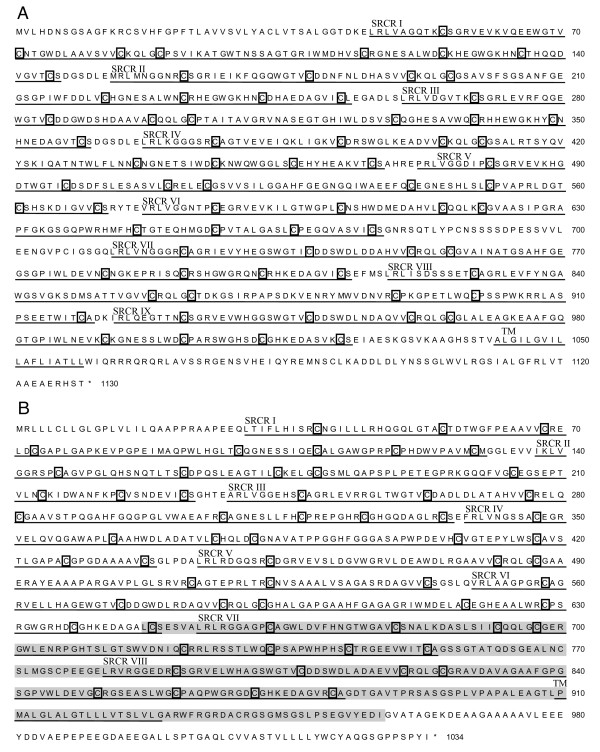
**Amino acid sequences of bovine CD163**. Translated amino acid sequence, based on cDNA sequences for (A) CD163A and based on annotated and determined cDNA (shaded) sequences for (B) CD163c-α. Individual SRCR domains, identified by comparison to consensus group B SRCR domain sequence, are underlined, domain numbers are indicated by roman numerals, and cysteines are boxed. Putative transmembrane regions (TM) were determined by the DAS transmembrane prediction server and are also underlined [70].

The CD163c-α amino acid sequence generated by automated prediction lacked a transmembrane region that was correlated with a gap in the genomic sequence at the expected location of the exon encoding the transmembrane domain (Fig. 1B). We investigated whether bovine CD163c-α has a transmembrane domain by amplifying and sequencing cDNA template from peripheral blood mononuclear cells using primers designed from known sequences in the genomic sequence, which bracketed the putative transmembrane region. We obtained transmembrane domain sequence in the same frame as known sequence upstream and downstream (Fig. 2B), indicating that the bovine CD163c-α is a transmembrane receptor. The bovine homologue of the human gene encoding CD163b was not found in the bovine genome assembly Btau 3.1, which could reflect its absence or a relatively large gap in the sequenced bovine genome.

### Bovine CD163 sequences

Based on the manual annotation of the bovine CD163c-α and CD163A gene sequences (Fig. 1), we designed primers to amplify CD163A and CD163c-α transcripts. Deduced amino acid sequences based on the obtained cDNA sequence of CD163A (Fig. 2A) and on both the annotated genomic sequence and obtained cDNA sequence (shaded) of CD163c-α (Fig. 2B) were used to evaluate their predicted protein structures. Bovine CD163A has nine SRCR domains and bovine CD163c-α has eight SRCR domains in their extracellular regions, which are underlined in the predicted protein (Fig. 2). Most of the SRCR domains conform to the eight-cysteine consensus of SRCR group B. The exceptions are the eighth SRCR domain of bovine CD163A and the fifth SRCR domain of bovine CD163c-α. These SRCR domains lack the second and seventh cysteines, which form a disulfide bond in most, but not all, other SRCR group B domains. Estimates of the evolutionary divergence between SRCR domain amino acid sequences confirm that bovine CD163A is most similar to human CD163A (Table 1) and that bovine CD163c-α is most similar to human CD163c-α (Table 2) in both the order of SRCR domains and sequence identity. Bovine CD163A is identical to human CD163A in its SRCR domain organization, and highly similar in its sequence with 82% overall sequence identity. Bovine CD163c-α is identical to human CD163c-α in its SRCR domain organization and 68% identical in overall sequence. The evolutionary divergence between bovine CD163A or bovine CD163c-α SRCR domains and bovine WC1 SRCR domains is greater (Table 3), indicating that the genes we have identified as bovine CD163A or bovine CD163c-α are not WC1 genes. The sequences of the cytoplasmic domains of CD163c-α and CD163A from cattle do not exhibit significant sequence identity to each other (data not shown), although they contain the tyrosine-based motifs of YEDI and YREM respectively.

**Table 1:** Estimates of evolutionary divergence between human CD163A, bovine CD163A and bovine CD163c-α SRCR domains

			HsCD163A								
	SRCR domain		h	i	j	k	b	c	d	e	d'
			1	2	3	4	5	6	7	8	9
**BtCD163A**	**h**	**1**	**0.179**	**0.462**	**0.410**	0.538	0.603	0.641	**0.474**	0.679	0.513
			(0.043)	(0.056)	(0.056)	(0.056)	(0.055)	(0.057)	(0.057)	(0.053)	(0.057)
	**i**	**2**	**0.436**	**0.167**	0.513	0.551	0.564	0.628	**0.436**	0.692	0.500
			(0.056)	(0.042)	(0.057)	(0.056)	(0.056)	(0.056)	(0.056)	(0.053)	(0.057)
	**j**	**3**	**0.372**	0.500	**0.115**	0.590	0.628	0.615	**0.449**	0.705	0.500
			(0.055)	(0.057)	(0.036)	(0.056)	(0.055)	(0.056)	(0.056)	(0.052)	(0.057)
	**k**	**4**	0.590	0.590	0.590	**0.115**	0.692	0.654	0.526	0.731	0.551
			(0.056)	(0.056)	(0.056)	(0.036)	(0.052)	(0.056)	(0.057)	(0.053)	(0.056)
	**b**	**5**	0.577	0.513	0.628	0.641	**0.090**	0.538	0.564	0.731	0.603
			(0.056)	(0.057)	(0.055)	(0.054)	(0.032)	(0.056)	(0.056)	(0.050)	(0.055)
	**c**	**6**	0.590	0.628	0.615	0.679	0.526	**0.167**	0.577	0.782	0.577
			(0.056)	(0.057)	(0.056)	(0.053)	(0.057)	(0.042)	(0.056)	(0.047)	(0.056)
	**d**	**7**	0.526	**0.474**	**0.474**	0.551	0.577	0.551	**0.090**	0.667	**0.474**
			(0.057)	(0.057)	(0.057)	(0.056)	(0.056)	(0.056)	(0.032)	(0.052)	(0.057)
	**e**	**8**	0.718	0.744	0.744	0.692	0.692	0.705	0.705	**0.231**	0.744
			(0.052)	(0.050)	(0.052)	(0.052)	(0.052)	(0.053)	(0.053)	(0.048)	(0.052)
	**d'**	**9**	0.551	0.538	0.551	0.526	0.603	0.538	**0.423**	0.692	**0.167**
			(0.056)	(0.056)	(0.056)	(0.057)	(0.055)	(0.056)	(0.056)	(0.052)	(0.042)

**BtCD163c-α**	**m**	**1**	0.705	0.718	0.679	0.615	0.718	0.692	0.641	0.808	0.615
			(0.052)	(0.051)	(0.053)	(0.055)	(0.051)	(0.052)	(0.054)	(0.045)	(0.055)
	**l**	**2**	0.667	0.718	0.679	0.692	0.654	0.667	0.692	0.821	0.718
			(0.053)	(0.052)	(0.052)	(0.052)	(0.054)	(0.053)	(0.050)	(0.043)	(0.051)
	**b**	**3**	0.577	0.564	0.641	0.590	**0.449**	0.577	0.538	0.731	0.551
			(0.056)	(0.056)	(0.054)	(0.056)	(0.056)	(0.056)	(0.056)	(0.050)	(0.056)
	**c**	**4**	0.667	0.654	0.628	0.628	0.615	0.526	0.590	0.731	0.615
			(0.053)	(0.054)	(0.055)	(0.055)	(0.055)	(0.057)0	(0.056)	(0.050)	(0.055)
	**n**	**5**	0.654	0.679	0.628	0.590	0.654	0.667	0.564	0.705	0.603
			(0.054)	(0.053)	(0.055)	(0.056)	(0.054)	(0.053)	(0.056)	(0.052)	(0.055)
	**d**	**6**	**0.474**	0.526	0.500	0.526	0.577	0.590	**0.397**	0.667	**0.436**
			(0.057)	(0.057)	(0.057)	(0.057)	(0.056)	(0.056)	(0.055)	(0.053)	(0.056)
	**e**	**7**	0.628	0.667	0.679	0.654	0.679	0.744	0.641	0.603	0.679
			(0.055)	(0.052)	(0.054)	(0.055)	(0.053)	(0.049)	(0.055)	(0.055)	(0.053)
	**d'**	**8**	0.538	0.526	0.538	0.538	0.564	0.628	**0.423**	0.654	**0.397**
			(0.056)	(0.057)	(0.056)	(0.056)	(0.056)	(0.055)	(0.056)	(0.054)	(0.055)

The number of amino acid differences per site from analysis between sequences is shown. All results are based on the pairwise analysis of 26 sequences. Standard error estimates are shown in parentheses and were obtained by using analytical formulas. Analyses were conducted using the amino p-distance model in MEGA4 [64]. All positions containing gaps and missing data were eliminated from the dataset (complete deletion option). There were a total of 78 positions in the final dataset. Estimates below 0.500 are in boldtype, and values on the diagonal corresponding to a high level of identity between sequences are underlined. SRCR domains are labelled with alphabetical designations as previously defined and by inferring the evolutionary relationships between SRCR domains using the Neighbor-Joining and Bayesian methods [1,65,69].

**Table 2:** Estimates of evolutionary divergence between human CD163c-α, bovine CD163A and bovine CD163c-α SRCR domains

		HsCD163c-α								
	SRCR domain		m	l	b	c	n	d	e	d'
			1	2	3	4	5	6	7	8
**BtCD163A**	**h**	**1**	0.577	0.692	0.590	0.641	0.641	0.500	0.628	0.513
			(0.056)	(0.052)	(0.056)	(0.054)	(0.054)	(0.057)	(0.055)	(0.057)
	**i**	**2**	0.590	0.731	0.551	0.628	0.667	**0.462**	0.641	**0.474**
			(0.056)	(0.050)	(0.056)	(0.055)	(0.053)	(0.056)	(0.054)	(0.057)
	**j**	**3**	0.615	0.679	0.654	0.628	0.641	**0.474**	0.628	0.526
			(0.055)	(0.053)	(0.054)	(0.055)	(0.054)	(0.057)	(0.055)	(0.057)
	**k**	**4**	0.551	0.654	0.615	0.692	0.628	0.577	0.679	0.590
			(0.056)	(0.054)	(0.055)	(0.052)	(0.055)	(0.056)	(0.053)	(0.056)
	**b**	**5**	0.654	0.667	**0.462**	0.590	0.679	0.538	0.692	0.564
			(0.054)	(0.053)	(0.056)	(0.056)	(0.053)	(0.056)	(0.052)	(0.056)
	**c**	**6**	0.654	0.654	0.551	0.500	0.667	0.590	0.731	0.615
			(0.054)	(0.054)	(0.056)	(0.057)	(0.053)	(0.056)	(0.050)	(0.055)
	**d**	**7**	0.615	0.615	0.551	0.590	0.577	**0.372**	0.679	**0.436**
			(0.055)	(0.055)	(0.056)	(0.056)	(0.056)	(0.055)	(0.053)	(0.056)
	**e**	**8**	0.718	0.795	0.744	0.756	0.679	0.654	0.577	0.692
			(0.051)	(0.046)	(0.049)	(0.049)	(0.053)	(0.054)	(0.056)	(0.052)
	**d'**	**9**	0.513	0.679	0.526	0.590	0.590	**0.436**	0.667	**0.410**
			(0.057)	(0.053)	(0.057)	(0.056)	(0.056)	(0.056)	(0.053)	(0.056)

**BtCD163c-α**	**m**	**1**	**0.462**	0.641	0.667	0.718	0.692	0.654	0.808	0.667
			(0.056)	(0.054)	(0.053)	(0.051)	(0.052)	(0.054)	(0.045)	(0.053)
	**l**	**2**	0.705	0.577	0.692	0.705	0.744	0.731	0.756	0.744
			(0.052)	(0.056)	(0.052)	(0.052)	(0.049)	(0.050)	(0.049)	(0.049)
	**b**	**3**	0.603	0.692	**0.167**	0.577	0.679	0.526	0.667	0.526
			(0.055)	(0.052)	(0.042)	(0.056)	(0.053)	(0.057)	(0.053)	(0.057)
	**c**	**4**	0.603	0.667	0.590	**0.218**	0.641	0.615	0.756	0.615
			(0.055)	(0.053)	(0.056)	(0.047)	(0.054)	(0.055)	(0.049)	(0.055)
	**n**	**5**	0.615	0.641	0.603	0.654	**0.231**	0.538	0.731	0.538
			(0.055)	(0.054)	(0.055)	(0.054)	(0.048)	(0.056)	(0.050)	(0.056)
	**d**	**6**	0.538	0.667	0.538	0.551	0.590	**0.218**	0.654	**0.410**
			(0.056)	(0.053)	(0.056)	(0.056)	(0.056)	(0.047)	(0.054)	(0.056)
	**e**	**7**	0.692	0.731	0.641	0.718	0.744	0.615	**0.179**	0.654
			(0.052)	(0.050)	(0.054)	(0.051)	(0.049)	(0.055)	(0.043)	(0.054)
	**d'**	**8**	0.551	0.692	**0.487**	0.577	0.564	**0.423**	0.628	**0.141**
			(0.056)	(0.052)	(0.057)	(0.056)	(0.056)	(0.056)	(0.055)	(0.039)

Analyses were conducted as described in Table 1.

**Table 3:** Estimates of evolutionary divergence between bovine archetypal WC1.1, bovine CD163A and bovine CD163c-α SRCR domains

			BtWC1.1					
	SRCR domain		a	b	c	d	e	d'
			1	2, 7	3, 8	4, 6, 9	5, 10	11
**BtCD163A**	**h**	**1**	0.551 (0.056)	0.667, 0.654 (0.053, 0.054)	0.641, 0.654 (0.054, 0.054)	**0.462, 0.462, **0.500 (0.056, 0.056, 0.057)	0.705, 0.692 (0.052, 0.052)	0.500 (0.057)

	**i**	**2**	0.526 (0.057)	0.577, 0.564 (0.056, 0.056)	0.590, 0.615 (0.056, 0.055)	**0.436, 0.410, 0.436 **(0.056, 0.056, 0.056)	0.705, 0.667 (0.052, 0.053)	**0.487 **(0.057)

	**j**	**3**	0.500 (0.057)	0.679, 0.654 (0.053, 0.054)	0.654, 0.679 (0.054, 0.053)	**0.449, 0.449, 0.449 **(0.056, 0.056, 0.056)	0.705, 0.705 (0.052, 0.052)	0.500 (0.057)

	**k**	**4**	0.564 (0.056)	0.654, 0.641 (0.054, 0.054)	0.615, 0.615 (0.055, 0.055)	0.564, 0.551, 0.577 (0.056, 0.056, 0.056)	0.705, 0.692 (0.052, 0.052)	0.500 (0.057)

	**b**	**5**	0.551 (0.056)	** 0.449, 0.436 **(0.056, 0.056)	0.577, 0.603 (0.056, 0.055)	0.526, 0.590, 0.513 (0.057, 0.056, 0.054)	0.718, 0.692 (0.051, 0.052)	0.538 (0.056)

	**c**	**6**	0.590 (0.056)	0.615, 0.590 (0.055, 0.056)	0.513, 0.538 (0.057, 0.056)	0.513, 0.590, 0.526 (0.057, 0.056, 0.057)	0.769, 0.731 (0.048, 0.050)	0.603 (0.055)

	**d**	**7**	**0.487 **(0.057)	0.641, 0.628 (0.054, 0.055)	0.590, 0.603 (0.056, 0.055)	**0.218, 0.321, 0.218 **(0.047, 0.053, 0.047)	0.667, 0.641 (0.053, 0.054)	**0.474 **(0.057)

	**e**	**8**	0.744 (0.049)	0.731, 0.705 (0.050, 0.052)	0.731, 0.731 (0.050, 0.050)	0.718, 0.744, 0.692 (0.051, 0.049, 0.052)	0.513, 0.500 (0.057, 0.057)	0.692 (0.052)

	**d'**	**9**	0.526 (0.057)	0.615, 0.590 (0.055, 0.056)	0.590, 0.590 (0.056, 0.056)	**0.423, 0.436, 0.397 **(0.056, 0.056, 0.055)	0.679, 0.667 (0.053, 0.053)	**0.359 **(0.054)

**BtCD163c-α**	**m**	**1**	0.654 (0.054)	0.756, 0.731 (0.049. 0.050)	0.679, 0.679 (0.053, 0.053)	0.654, 0.667, 0.654 (0.054, 0.053, 0.054)	0.795, 0.769 (0.046, 0.048)	0.628 (0.055)

	**l**	**2**	0.718 (0.051)	0.692, 0.705 (0.052, 0.052)	0.705, 0.718 (0.052, 0.051)	0.654, 0.654, 0.667 (0.054, 0.054, 0.053)	0.769, 0.795 (0.048, 0.046)	0.679 (0.053)

	**b**	**3**	0.551 (0.056)	0.538, 0.526 (0.056, 0.057)	0.577, 0.577 (0.056, 0.056)	0.513, 0.577, 0.526 (0.057, 0.056, 0.057)	0.718, 0.692 (0.051, 0.052)	0.526 (0.057)

	**c**	**4**	0.603 (0.055)	0.628, 0.615 (0.055, 0.055)	0.526, 0.538 (0.057, 0.056)	0.603, 0.615, 0.603 (0.055, 0.055, 0.055)	0.744, 0.744 (0.049, 0.049)	0.615 (0.055)

	**n**	**5**	0.513 (0.057)	0.679, 0.667 (0.053, 0.053)	0.654, 0.654 (0.054, 0.054)	0.564, 0.590, 0.564 (0.056, 0.056, 0.056)	0.744, 0.756 (0.049, 0.049)	0.564 (0.056)

	**d**	**6**	0.500 (0.057)	0.577, 0.564 (0.056, 0.056)	0.577, 0.590 (0.056, 0.056)	**0.346, 0.423, 0.333 **(0.054, 0.056, 0.053)	0.679, 0.667 (0.053, 0.053)	**0.487 **(0.057)

	**e**	**7**	0.705 (0.052)	0.667, 0.679 (0.056, 0.056)	0.692, 0.718 (0.052, 0.051)	0.628, 0.641, 0.628 (0.055, 0.054, 0.055)	0.526, 0.526 (0.057, 0.057)	0.615 (0.055)

	**d'**	**8**	0.526 (0.057)	0.577, 0.577 (0.053, 0.053)	0.615, 0.615 (0.055, 0.055)	**0.436, 0.423, 0.397 **(0.056, 0.056, 0.055)	0.615, 0.615 (0.055, 0.055)	**0.321 **(0.053)

Analyses were conducted as described in Table 1.

### Expression profiles of bovine CD163A and CD163c-α

**Figure 3:**
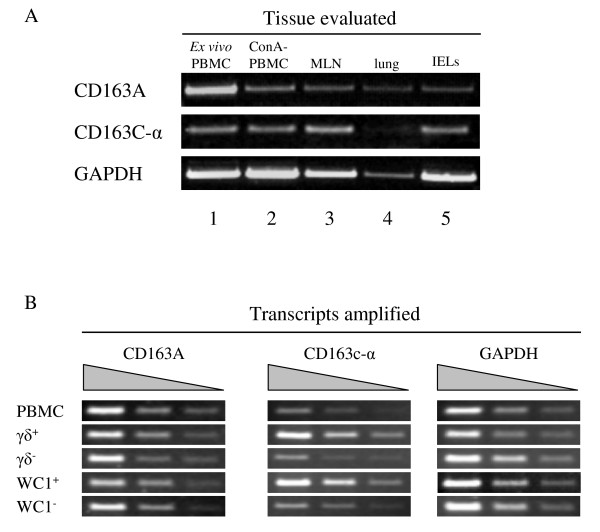
**Expression profiles of bovine CD163 genes**. (A) Bovine *ex vivo *PBMC (lane 1), ConA-activated PBMC (lane 2), mesenteric lymph node (MLN, lane 3), lung (lane 4) and intestinal epithelial lymphocytes (IELs, lane 5) were evaluated for expression of CD163A and CD163c-α by RT-PCR. GAPDH is shown for comparison. These results are representative of reactions (n = 3) performed for at least two animals. (B) Bovine total PBMC, γδ TCR^+ ^cells, γδ TCR ^- ^cells, WC1^+ ^cells, and WC1^- ^cells were evaluated for CD163A, CD163c-α, and GAPDH expression by RT-PCR. Templates were serially diluted by a factor of three. These results are representative of two reactions.

A variety of tissues were examined for expression of bovine CD163 transcripts including mesenteric lymph node (MLN), lung, intestinal intraepithelial lymphocytes (IELs, containing approximately 20% γδ T cells, data not shown) and peripheral blood mononuclear cells (PBMC). PBMC were evaluated both as *ex vivo *cells and following activation by Con A. Based on expressed sequence tag (EST) analysis in the NCBI UniGene database http://www.ncbi.nlm.nih.gov/sites/entrez?db=unigene, it was predicted that bovine CD163c-α is expressed in the intestine and that bovine CD163A is expressed in the intestine and mesenteric lymph node (MLN). Interestingly, we found CD163A to be expressed in all tissues evaluated (Fig. 3A) and not just in intestine and MLN. Surprisingly, we found that CD163A is also expressed on WC1^+ ^and γδ TCR^+ ^as well as WC1^- ^and γδ TCR^- ^cells sorted from *ex vivo *PBMC (Fig. 3B) indicating that the expression of bovine CD163A is not restricted to monocytes and macrophages, as is the case in humans [5,41].

We found that CD163c-α transcripts were relatively higher in PBMC, MLN and IELs, than in lung (Fig. 3A), consistent with expression in leukocytes. The CD163c-α family member SCART2 has been shown to be expressed in murine γδ T cells [25]. To test the hypothesis that bovine CD163c-α is expressed on a T cell subset separate from the WC1^+ ^γδ T cell subset, we evaluated the expression of bovine CD163c-α on WC1^+^, WC1^-^, γδ TCR^+ ^and γδ TCR^- ^cells sorted from *ex vivo *PBMC (Fig. 3B). We found that CD163c-α was preferentially expressed in the WC1+ or γδ TCR+ cell populations in PBMC, suggesting that it increases the potential receptor repertoire of WC1^+^γδ T cells.

### Analysis of CD163 and WC1 SRCR domains

**Figure 4:**
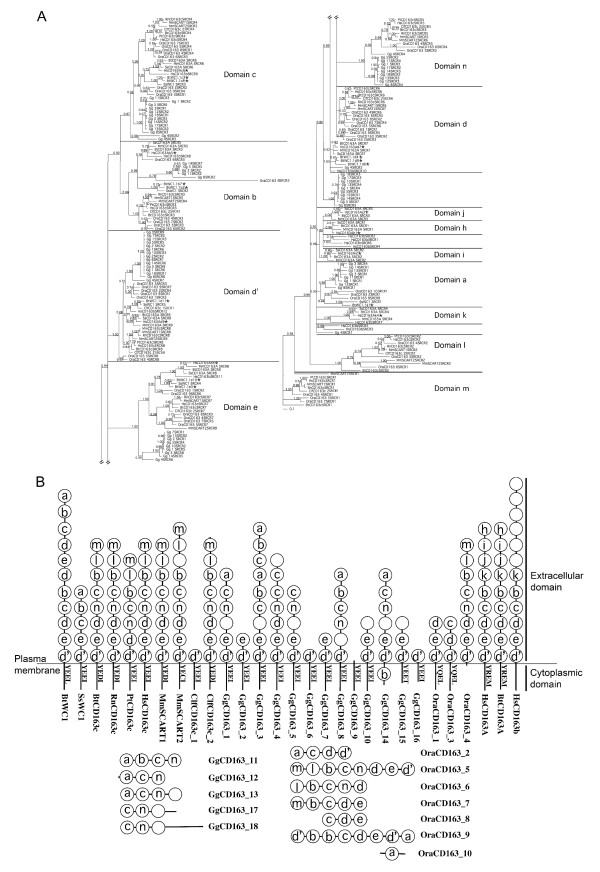
**CD163 family member SRCR domain organization and cytoplasmic tyrosine-based motifs**. >(A) CD163 family members from multiple species were identified by BLAST searches with bovine WC1, CD163A and CD163c-α SRCR and cytoplasmic domains. The evolutionary history of 242 taxa was inferred, using MrBayes3.2 to approximate the posterior probabilities of trees, shown at branch nodes [65]. SRCR domains clustering in a clade with pre-defined SRCR domains (asterisks) were identified and SRCR domains clustered together, but not in association with known SRCR domains, were assigned a new letter designation [1]. *Rattus norvegicus *(Rn) CD163c-α, *Pan troglodytes *(Pt) CD163c-α, *Canis lupus familiaris *(Clf) CD163c_1 and ClfCD163c_2, *Gallus gallus *(Gg) CD163_1 through GgCD163_18, and *Ornithorhynchus anatinus *(Ora) CD163_1 through OraCD163_10 are predicted from genomic sequence; *Bos taurus *(Bt) WC1, *Sus scrofa *(Ss) WC1, *Homo sapiens *(Hs) CD163c-α (partial cDNA, corrected from genomic sequence), *Mus musculus *(Mm) SCART1, MmSCART2, HsCD163A and HsCD163b cDNA have been isolated. (B) Summary of CD163 family member SRCR domain organization and cytoplasmic tyrosine based motifs. SRCR domain assignments were made as shown in Fig. 4A, with confirmation by analysis utilizing the Neighbor-Joining algorithm with the JTT model (data not shown). CD163 family members with transmembrane domains are shown with the plasma membrane and location of the transmembrane domains represented by the horizontal line. Tyrosine-based signalling motifs similar to that found in BtWC1 are shown [35]. CD163 family members without transmembrane domains are shown horizontally, with the N-terminus on the left.

We undertook a phylogenetic analysis of CD163 family members and their closest molecular relative, WC1, with the goal of understanding the evolution and function of WC1 and CD163 family members. We performed multiple alignments of individual SRCR domains in bovine CD163c-α and bovine CD163A with defined SRCR domains from bovine WC1 and human CD163A [1]. The SRCR domains from bovine WC1 and human CD163A have previously been assigned an alphabet letter designation "a", "b", "c", "d", "d' ", "e", "h", "i", "j", or "k" [1]. SRCR domains in bovine CD163c-α and bovine CD163A that clustered in the same clades as these previously designated SRCR domains (Fig. 4A, indicated by asterisks) were assigned the same designation. SRCR domains that clustered in separate clades from the clades containing already designated SRCR domains were assigned the new alphabet letter designations "m", "n" and "l" (Fig. 4A,4B). Phylograms obtained using Bayesian analysis (Fig. 4A) showed that the first SRCR domain of bovine CD163c-α (BtCD163cSRCR1) and the first SRCR domains of CD163c-α-like molecules from the duck-billed platypus (OraCD163_4SRCR1, OraCD163_5SRCR1 and OraCD163_7SRCR1) are in the same clade as the first SRCR domain of primate, rodent and canine CD163c-α (designated "m"). The clade containing the fifth SRCR domain (designated "n") in bovine CD163c-α also includes SRCR domains from CD163c-α-like molecules from eutherian mammals, the duck-billed platypus and the chicken (Fig. 4A). The second SRCR domain of bovine CD163c-α (BtCD163cSRCR1) clusters in the same clade as other SRCR domains designated "l" from eutherian mammals and the duck-billed platypus (Fig. 4A). SRCR domains that we have designated as "d" are clustered in sister clades to defined "d" SRCR domains by Bayesian analysis: however, their designation as domain "d" is also supported by their position in the protein in reference to other SRCR domains (Fig.4B). Several SRCR domains from chicken CD163 molecules clustered apart from the "d" SRCR domain; although, their identification as "d" SRCR domains is suggested by their position in the protein (e.g. GgCD163_1 SRCR4). These SRCR domains were left undesignated (Fig. 4A,4B). The first six SRCR domains of human CD163b do not reproducibly cluster in the same clade as other defined SRCR domains and were also left undesignated (Fig. 4A,4B). Notably, CD163b has thus far been found only in primates and horses. It is also of note that SRCR domain "k", which appears to be diagnostic of CD163A or CD163b, does not appear in any of the annotated genes from the duck-billed platypus or the chicken, suggesting that CD163A or CD163b is not encoded in these animals' genomes. Thus, there appear to be at least 4 types of CD163 family members, not all of which appear in every species, and some of which are duplicated within a species: CD163A, CD163b, CD163c-α, and WC1.

Most of the CD163 molecules, especially those with a transmembrane domain, contain the usually membrane proximal "d' " and "e" SRCR domains (Fig. 4B). These domains from chicken and duck-billed platypus cluster together within each species, whereas CD163A "d' " and "e" SRCR domains from eutherian mammals are found in separate sub-clades from CD163b, CD163c-α, and WC1 "d' " and "e" SRCR domains from eutherian mammals (Fig. 4A).

### Relationship of CD163c-α and WC1

The relationship of CD163c-α to WC1 is particularly intriguing since WC1 expression is restricted to γδ T cells and CD163c-α expression is enriched in WC1+γδ T cells (Fig. 3B). When we performed a multiple SRCR domain alignment and phylogenetic tree analysis of CD163 proteins across multiple species, we found that the SRCR domain structure and organization of bovine CD163c-α (m-l-b-c-n-d-e-d') is most similar to CD163c-α from other eutherian mammalian species, such as human, chimpanzee, dog, and mouse and rat. The SRCR domains "m" and "l" and "n" were unique to CD163c-α molecules and were not found to occur in bovine or swine WC1, nor did they occur in the other CD163 family members CD163A or human CD163b (Fig. 4A,4B). Domains "m", "l", and "n" clustered in separate clades than the WC1 domain "a", as well as from the first six SRCR domains from CD163b and SRCR domains "h", "i", "j", and "k" from CD163A (Fig. 4A,4B).

The chicken and the duck-billed platypus possess more CD163 genes than primates, dogs or rodents, displaying a diverse repertoire more similar to that seen with WC1 genes in the artiodactyls. The chicken possesses at least eighteen CD163 genes while the duck-billed platypus has at least ten CD163 genes. Seven of the chicken CD163 genes and three of the duck-billed platypus CD163 genes contained SRCR domains that clustered in the clade containing WC1 SRCR domain "a" (Fig. 4A). This is notable because domain "a" is the source of most of the diversity in WC1 family members and is thus thought to be the most likely SRCR domain to be responsible for WC1 isoform-specific function [31]. Chicken CD163_3 and platypus CD163_2 were the closest to bovine WC1 in extracellular SRCR organization, with SRCR domain organization of (a-b-c-a-b-c-x-e-d') and (a-c-d-d'), respectively, compared to the bovine WC1 SRCR domain organization of (a-b-c-d-e-d-b-c-d-e-d') (Fig. 4B). Six chicken CD163 proteins show a hybrid SRCR domain organization that is intermediate between WC1 and CD163c-α, with the SRCR domain "a" diagnostic of WC1 and SRCR domain "n" diagnostic of CD163c-α. For example, chicken CD163_1 and CD163_8 possess a N-terminal domain "a" characteristic of WC1 but an SRCR domain cassette (c-n-d-e-d') characteristic of CD163c-α. In contrast, CD163 molecules from the duck-billed platypus contain either the WC1- diagnostic domain "a", or CD163c-α-diagnostic domains "m","l", or "n", but not both.

The consensus group B SRCR domain contains eight cysteines, with disulfide bonds formed between the first and fourth, the second and seventh, the third and eighth, and the fifth and sixth cysteines [42]. Some of the SRCR domains lack the second and/or the seventh cysteine, which results in a predicted SRCR domain with only three disulfide bonds rather than four disulfide bonds. However, MmSCART2_SRCR3 (missing C2, C4 and C7), Gg_1SRCR2 (missing C5, C7, and C8), Gg_4SRCR7 (missing C7 and C8), Gg_5SRCR7 (missing C7 and C8), OraCD163_3SRCR2 (missing C6 and C7), OraCD163_7SRCR4 (missing C1), OraCD163_9SRCR2 (missing C2, C6, and C7), and OraCD163_6SRCR4 (missing C2, C6, and C7) are missing additional cysteines resulting in SRCR-like domains with only one to three potential disulfide bonds. It is not known how these changes will affect SRCR domain structure and function.

### Relationship of cytoplasmic sequences

**Figure 5:**
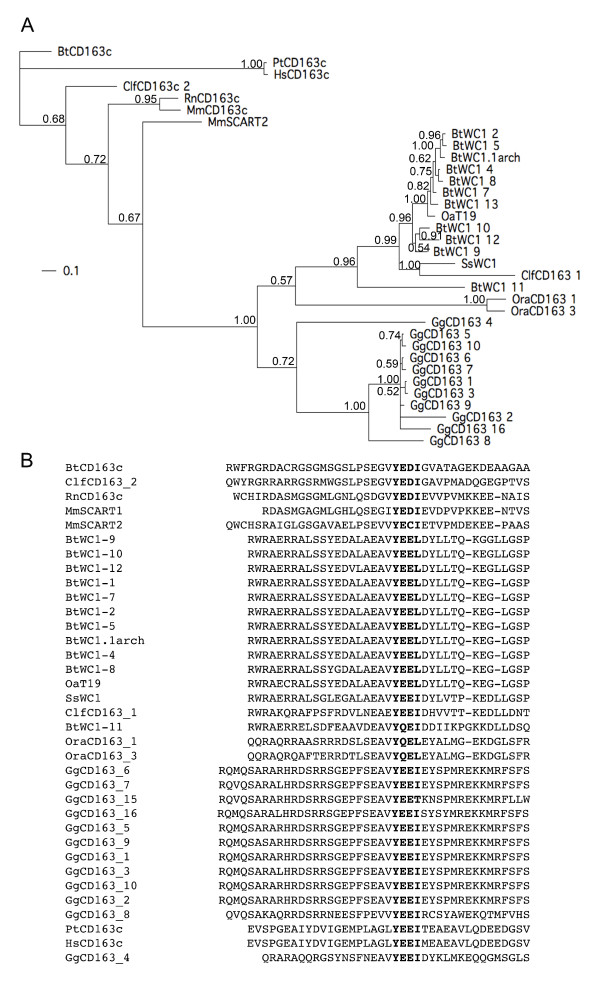
**WC1 and CD163cα tyrosine-based motifs in the cytoplasmic domain**. (A)	Cytoplasmic domains were determined by ascertaining the transmembrane domain, using the DAS transmembrane server [70]. Proteins are labelled as described in Fig. 4, with the addition of bovine WC1 cytoplasmic domains [31]. The evolutionary history of 34 taxa was inferred using Bayesian analysis in MrBayes3.2 [65]. Markov chain Monte Carlo analysis was performed for 830,000 cycles, using 2 runs of 4 chains each, a temperature setting of 0.2, and an amino acid mixed model to approximate the posterior probabilities of trees, shown at branch nodes. The average standard deviation of split frequencies was 0.01, which was diagnostic of convergence at < 0.05. (B)	Multiple alignment of the amino acids surrounding the tyrosine motif phosphorylated in WC1 (bold) with similar amino acid sequences from CD163 family members [35].

Several of the WC1-like or CD163-α-like molecules from chicken or duck-billed platypus do not have transmembrane or cytoplasmic domains; although, they are predicted open reading frames from genomic sequence and may not be complete. The cytoplasmic domain of chicken CD163_14, defined as being C-terminal to a transmembrane domain, is unusual in that it is not similar in sequence to other CD163 family members, but instead contains a SRCR domain (Fig. 4B). The cytoplasmic domains of bovine or human CD163A or human CD163b do not have any significant sequence identity with the cytoplasmic domains of CD163c-α-like or WC1-like molecules; although bovine and human CD163A have the tyrosine-based motif YREM in their cytoplasmic domains (Fig. 4B). We performed multiple amino acid alignment and phylogenetic tree analysis of the other cytoplasmic domains of CD163c-α-like and WC1 molecules and found that the cytoplasmic domains from canine ClfCD163_1, chicken and platypus CD163 molecules cluster more closely with the cytoplasmic domains of bovine and swine WC1, than with the cytoplasmic domains of canine ClfCD163_2, primate, bovine, and rodent CD163c-α (Fig. 5A).

Strikingly, all CD163c-α-like molecules from primate, canine, rodent, bovine, monotreme and bird species share a Y-(Q/E)-(D/C/E)-(I/L) motif with WC1, with the exception of chicken CD163_15, which has a threonine in place of the isoleucine or leucine (Fig. 5B). Phosphorylation of the YEEL tyrosine motif in WC1 is required for its potentiation of T cell activation, suggesting that WC1 and CD163c-α may signal via the same mechanism [35].

## Discussion

**Figure 6:**
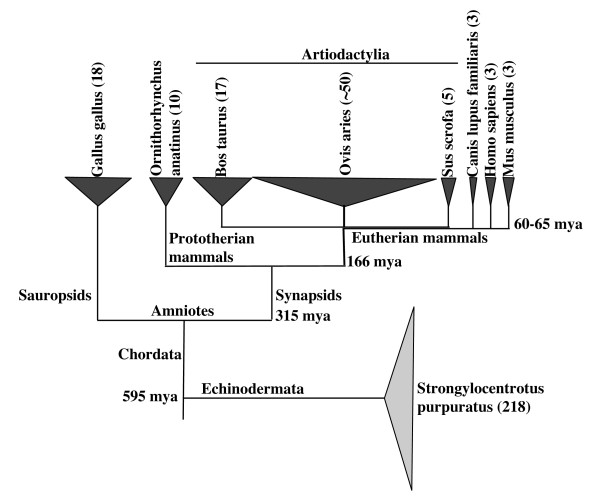
**CD163 family members in multiple species**. Numbers of group B SRCR CD163 family members in a representative of the sauropsid lineage, a prototherian mammal, and eutherian mammals are shown. The number of group A SRCR domain molecules in the echinoderm *Strongylocentrotus purpuratus *are shown [71]. Echinoderms diverged from chordates 595 million years ago; other divergence times are described on the Tree of Life web project http://tolweb.org[72].

Many genes that have been predicted to belong to the CD163 family have been erroneously classified as CD163A or CD163b homologues in their Entrez Gene reports. For example, ClfCD163_2, RnCD163c-α and MmSCART1 are referred to as similar to M160 or *CD163L1*, which are alternative names for CD163b, despite their greater similarity to CD163c-α in their SRCR identity and organization and in their cytoplasmic domain sequence. GgCD163_10 is referred to as CD5-like, despite showing greater sequence similarity to CD163c-α than CD5. Other genes from the chicken and platypus that are most similar to CD163c-α and WC1 are referred to as similar to CD163v2 and CD163v3, which are names for alternatively spliced isoforms of CD163A. It has been speculated that the plethora of CD163 molecules in the platypus are CD163A homologues important for regulating blood serum levels of free hemoglobin, instead of CD163c-α or WC1 homologues, which are more likely to be involved in the immune response [43]. In this study, we show that these genes encode CD163c-α, WC1, or CD163c-α/WC1-like proteins, based on the sequence of their SRCR and/or cytoplasmic domains. Genes coding for WC1-like proteins in non-artiodactyls have not been previously identified. SRCR domains "e " and "d' " (Fig. 4A) and cytoplasmic domains (Fig. 5A) that are common to many CD163 molecules across species cluster in sub-clades characterized by eutherian mammalian, platypus or chicken origin, suggesting that gene conversion in multi-gene families over millions of years of evolution has driven homogeneity [44]. Artiodactyls, platypus and chicken all display an expansion of WC1/CD163c-α-like genes (Fig. 6). The platypus and the chicken do not have a CD163A gene, suggesting that CD163A has evolved since the divergence of eutherian mammals from monotremes. In contrast, eutherian mammals have one CD163A gene and non-artiodactyl eutherian mammals have a maximum of two CD163c-α genes. This suggests that expression of multiple WC1/CD163c-α genes is the ancestral state and that eutherian mammals other than artiodactyls have lost multiple WC1/CD163c-α genes, since mammals diverged from the sauropsid lineage leading to birds and reptiles 315 million years ago [43]. In a similar finding, the platypus, sheep and cow share an expansion of the cathelicidin antimicrobial peptide gene family, whereas primates and rodents have only a single cathelicidin gene [45]. The primate or rodent cathelicidin must function equivalently to the plethora of cathelicidins in the platypus or ruminants; analogously, the one or two CD163c-α molecules in non-artiodactyl eutherian mammals may fulfil a function like that of WC1, in regulating the γδ T cell response.

Chickens, cattle and sheep are known to have a high percentage of γδ T cells in their peripheral blood. They are referred to as "γδ T cell high" species. In contrast, mice and humans have lower numbers of γδ T cells in their peripheral blood and are referred to as "γδ T cell low" species [46]. It is not known whether the duck-billed platypus is a γδ T cell high or γδ T cell low species, but it does possess γδ T cells [47]. We show that the chicken and platypus have multiple CD163c-α/WC1-like genes, a circumstance that is similar to the multiple WC1 genes in the genomes of the artiodactyls. We hypothesize that a diverse array of CD163c-α/WC1 SRCR transmembrane receptors conveys a selective advantage to the immune response to pathogens by γδ T cells and that WC1 and CD163c-α genes have been conserved because they play an important role in the response to pathogens.

Phosphorylation of the second tyrosine in the WC1 cytoplasmic domain is required for WC1-mediated potentiation of T cell activation though the TCR, thus, the conservation of this motif in WC1-like and CD163c-α-like genes over millions of years of evolution supports our hypothesis that CD163c-α plays a role similar to WC1 as a co-receptor to an activating receptor [35]. In contrast, it is unknown if the tyrosine-based motif YREM in CD163A of human or cattle is a phosphorylation target or whether it participates in membrane proximal signaling by CD163A after ligation of this receptor. Phosphorylation of CD163A by the serine/threonine kinases casein kinase II and protein kinase C-α (PKC-α) is tied to cytokine production induced by CD163A cross-linking [19]. Detection of transcripts for bovine CD163A in sorted WC1 ^+ ^and γδ^+ ^T cells, as well as in tissues containing monocytes and macrophages, suggests that bovine CD163A may play a role in the regulation of the γδ^+ ^T cell immune response, either through activation of transmembrane CD163A or proteolytic cleavage and release of soluble CD163A.

The characterization of the extracellular SRCR structure of WC1 and CD163 family members may help to isolate their ligands, which in turn would help us to better understand their role in the γδ T cell response. The role of WC1 and the proposed role for CD163c-α are consistent with other group B SRCR family transmembrane receptors expressed on T cells, such as CD5 and CD6. CD6 binds to the protein ALCAM/CD166 [48]. CD5 has been shown to bind to various proteins, including CD72, a 40-80 kDa glycoprotein expressed on murine B splenocytes and activated T cells, a human 150 kDa glycoprotein expressed on peripheral blood monocytes, certain IgV_H _framework sequences and a bovine 200 kDa protein expressed on activated B cells [49-54]. Ligation of CD5 or CD6 affects responses through the T cell receptor and B cell signaling and lymphocyte activation [55,56]. It is known that SRCR molecules also bind to molecules other than proteins; for example, the group B SRCR molecules Spα, CD6, DMBT1 and CD163A bind to bacteria and Spα and CD6 specifically bind to the bacterial products lipoteichoic acid (LTA) and lipopolysaccharide (LPS) [1,13,14,39,57]. The SRCR domains in WC1 or bovine CD163c-α do not have the bacteria-binding motif found in multiple SRCR domains of DMBT1 [58]. However, the requirement for expression of specific WC1 receptors for the γδ T cell response to the bacteria *Leptospira *(Wang F, Herzig CTA, Hsu H, Chen C, Baldwin CL, Telfer JC: Scavenger receptor WC1 contributes to the γδ T cell responses to *Leptospira*, submitted) suggests that WC1/CD163c-α could recognize pathogen-associated molecules or a protein whose expression is induced by exposure to *Leptospira. *Although the ligands for WC1 and CD163c-α are unknown, domains 9 (d) and 11 (d') of bovine WC1 interact with an unknown molecule on the surface of macrophages or dendritic cells [59]. In contrast, another CD163 family member, CD163A, is known to bind haptoglobin-hemoglobin complexes and TWEAK through its third SRCR domain ("j") and to bacteria or a molecule on erythroblasts via its second SRCR domain ("i") [10-13]. Neither of these domains occurs in either WC1 or CD163c-α, suggesting that CD163c-α ligation could be mediated by its unique domains "m", "l", or "n", and that it does not bind to haptoglobin-hemoglobin complexes. Since most of the diversity of bovine WC1 is found in domain "a", it is a reasonable candidate for the ligand-binding domain of the WC1 multi-gene family, which confers diversity on the γδ T cell response to pathogens.

## Conclusions

We have annotated the genes encoding CD163c-α and CD163A in cattle and shown that bovine CD163A is expressed in tissues containing monocytes and macrophages, as well as in sorted γδ T cells. The expression of bovine CD163c-α is enriched in WC1^+ ^γδ T cells and it shares the conserved tyrosine motif necessary for WC1 signalling in T cells, implying that it functions similarly to WC1 in acting as a co-receptor for the γδ TCR. We have characterized CD163c-α and WC1/CD163c-α gene products in multiple species, correcting the incorrect assignment of many of them as CD163A gene products. The expansion of WC1, CD163c-α and WC1/CD163c-α genes is correlated with a high level of γδ T cells in the peripheral blood of species separated by approximately 300 million years of evolution, suggesting that a diverse array of these molecules conveys a selective advantage to the γδ T cell response. Future studies will address the significant questions of both the signalling potential of CD163c-α and CD163A in γδ T cells and the identification of ligands for CD163c-α/WC1 molecules.

## Methods

### Genome annotation

In conjunction with the Bovine Genome Sequencing Consortium http://genomes.arc.georgetown.edu/drupal/bovine/, manual annotation of the CD163 genes was performed using the Apollo Genome Annotation and Curation Tool, version 1.6.5 and the bovine genome assembly Btau_3.1 [60]. Putative CD163 genes were identified by performing a BLAST search with orthologous mouse and human genes against the Bovine Official Gene Set (called GLEAN). The predicted gene models identified from the search included GLEAN_00453 for CD163A and GLEAN_14183 for CD163c-α. These were analyzed using the Apollo software and annotated based on available EST evidence and BLAST search results.

### Sequence analyses

Nucleotide sequences were aligned and consensus sequences were created using BioEdit version 7.0.5.3 [61]. Exon/intron structure schematics were based on alignments of cDNA and genomic DNA sequence using SIM4 and visualization with LalnView http://pbil.univ-lyon1.fr/software/lalnview.html[62].

Amino acid sequences used for comparison in phylogenetic analyses and their accession numbers are described in Additional file 1, Table S1. SRCR domains were identified according to the group B SRCR consensus and compared to HsCD163A, HsCD163c-α, BtWC1, HsDBMT1, HsCD5, and HsCD6 [42]. Molecules containing group B SRCR domains clustering with CD163 family members were defined as CD163 family members; molecules containing group A SRCR domains or SRCR group B domains clustering with HsDBMT1, HsCD5 and HsCD6 were excluded from analysis. Genomic WC1 cytoplasmic domain sequences used for comparison here have been previously described [31]. Multiple sequence alignments were performed using Clustal X 2.0.8 [63]. Pairwise and multiple alignment penalties for gaps were 10.0, 0.10 for gap extension and Gonnet 250 for protein weight matrix. Alignments were refined in MacClade 4.08. Phylogenetic trees were created using the Neighbor-Joining algorithm in MEGA4 and Bayesian analysis in MrBayes3.2 [64,65]. For Bayesian analysis, 2 runs with 3 cold chains and 1 heated chain each were done. An amino acid mixed model was used to approximate the posterior probabilities of trees. The 242-taxa SRCR domain alignment was run independently three times, with temperature settings of 0.15, 0.2, and 0.25, for 2.6, 2.0, and 2.5 million generations respectively. Trees were sampled every 100 generations and the burnin fraction was 0.5. The convergence diagnostic used was the average standard deviation of split frequencies, which were < 0.05 (0.0427, 0.031, and 0.030) for the three runs. The consensus trees from the three runs had the same overall topology. The consensus tree from the run set to temperature 0.25 and 2.5 million generations (average standard deviation of split frequencies 0.030, average potential scale reduction factor 1.016) is shown in this paper. The 34-taxa cytoplasmic domain alignment was run once with a temperature setting of 0.2 for 830,000 generations. Trees were sampled every 100 generations and the burnin fraction was 0.25. The average standard deviation of split frequencies was 0.01 and the average potential scale reduction factor was 1.003. Phylograms were visualized using TreeView X 0.5.0 [66].

### Animals, cells and tissues

Belted Galloway cattle of between 1 and 2 years of age were housed at the University of Massachusetts Amherst according to institutional and USDA guidelines. Animal protocols were approved by the University of Massachusetts Amherst institutional animal care and use committee. Blood was obtained via jugular venipuncture and collected into a solution of heparin. Peripheral blood mononuclear cells (PBMC) were isolated from blood via density gradient centrifugation over Ficoll-Paque^TM ^PLUS (GE Healthcare Bio-Sciences, Piscataway, NJ) by standard techniques. Culture medium consisted of RPMI 1640 (Invitrogen, Carlsbad, CA), 10% heat-inactivated FBS, 2 mM L-glutamine, 50 μg/ml gentamicin, and 50 μM 2-ME. PBMC were cultured at 2.5 × 10 ^6 ^cells/ml with Concanavalin (ConA 1.0 μg/ml; Sigma-Aldrich, St. Louis, MO) at 37°C with 5% CO_2 _in air for 3 days where indicated. For evaluation of CD163 expression in intestine, intraepithelial lymphocytes (IELs) were isolated from the ileum of two-month old cattle using nonenzymatic methods [67]. IELs were stained for cell surface differentiation molecules, fixed in 1% paraformaldehyde, and analyzed via flow cytometry (LSR II, BD Biosciences, San Jose, CA) using the following primary monoclonal antibodies (mAbs): CC15 (pan-WC1; Serotec, Raleigh, NC), GB21A (δ TCR; VMRD, Pullman, WA), and IL-A12 (CD4;) [68]. Secondary antibodies used were isotype-specific polyclonal goat anti-mouse Ig conjugated with fluorescein isothiocyanate (FITC) (Southern Biotechnology, Birmingham, AL). Flow cytometric data was analyzed using FlowJo version 7.2.2 (Tree Star, Ashland, OR).

Purified γδ T cells and WC1 ^+ ^γδ T cells were obtained from ex vivo PBMC by magnetic bead sorting (MACS; Miltenyi Biotec, Auburn, CA). Cells were suspended in PBS containing 6% heat-inactivated horse serum and incubated with monoclonal antibodies (mAbs) GB21A or CC15 for 20 min on ice, washed twice with PBS containing 0.5% bovine serum albumin and 2 mM EDTA and resuspended in the same along with goat anti-mouse IgG microbeads (Miltenyi Biotec) following the manufacturer's instructions. Cells were purified over pre-cooled MS-separation columns (Miltenyi Biotec); the positive and negative fractions were collected and each was passed over an additional separation column in order to optimize purity. Purity was assessed by flow cytometry and was found to be 99.6% for γδ T cells, 98.1% for γδ T cell-depleted cells, 95.5% for WC1^+ ^γδ T cells and 92.7% for WC1^+ ^γδ T cell-depleted cells.

### RNA isolation and RT-PCR

Pelleted *ex vivo *or ConA-activated PBMC, sorted cells, and IELs were resuspended in TRIzol (Invitrogen). MLN and lung tissue samples frozen in TRIzol were thawed and homogenized. RNA was isolated according to the manufacturer's protocol. For RNA isolation from sorted cells, glycogen (Invitrogen) was added to the aqueous phase prior to RNA precipitation. Reverse transcription (RT) was performed using 1 μg of total RNA, oligo dT primers and AMV reverse transcriptase (AMV RT kit; Promega, Madison, WI). Prior to RT reactions, RNA derived from MLN and lung was treated using RQ1 RNase-Free DNase (Promega) according to the manufacturer's protocol. 2 μl of cDNA was used as template in subsequent PCR reactions.

Primer pair sequences used to obtain CD163A and CD163c-α cDNA sequences are as follows: (1) CD163Autr-for 5'-GAG TGG ACA AAC TCA GAA TGG TG and CD163A-rev2 5'-GAG GAA TTA TAT AGG TCC AGA TCA TC; (2) CD163A2952-for 5'-CAT ATG GCT CAA TGA AGT GAA GTG and CD163Autr-rev 5'-GTG CAT CAC AGG CTT CTT ATT ATG; (3) CD163c2312-for 5'-GTG TGG AGC TCT GGC ACG CTG and CD163c-rev2 5'-CAA TGT CCT CAT AAA CAC CTT CTG; (4) CD163c1938-for 5'-CCT CTG CTC AGA GTC AGT G and CD163c2312-rev 5'-CAG CGT GCC AGA GCT CCA CAC using PCR Master Mix (Promega). Cycling parameters were 30 s at 94°C, 30 s at 50°C and approximately 1 min/kb at 68°C for 35 cycles. PCR products were ligated in the pCR2.1 vector (Invitrogen) and sequenced (GeneWiz, South Plainfield, NJ) using the T7 forward and M13 reverse primers, as well as the following sequence specific primers, where applicable: CD163Aseq2-for 5'-CCA ATC TGG TTT GAT GAT CTG GTA; CD163Aseq3-for 5'-TCT GAC TTC TCT CTG GAA TCG; CD163Aseq4-for 5'-CAG AAC TGC AGG CAT AAG GAG; CD163Aseq1-rev 5'-GCT GCC CCA AGC TCC GTT G; CD163Aseq2-rev 5' - CAT TCG TGA TGT CTG CAC TG; CD163A1824-rev 5'-CAT GTC CCA GTG AGA GTT GCA GAG.

For expression analysis PCR Master Mix (Promega) was used. Primer sequences were as follows: GAPDH-for 5'-GTCATCATCTCTGCACCTTCT; GAPDH-rev 5'-ACCACCTTCTTGATCTCATCAT; For CD163A and CD163c-α primer pairs (2) and (3) described above were used, respectively. Cycling parameters were 30 s at 94°C, 30 s at 55°C and 1 min at 68°C for 30 cycles (GAPDH) or 35 cycles (CD163A and CD163c). CD163A and CD163c-α PCR products were directly sequenced (GeneWiz, South Plainfield, NJ) in order to verify primer specificity.

## Authors' contributions

CTAH annotated the bovine CD163 genes, performed the expression analysis and helped prepare the manuscript. WRW supplied the bovine tissues used for the expression analysis. CLB helped in the study coordination and manuscript preparation. JCT conceived of the study, performed the phylogenetic analysis and drafted the manuscript. All authors read and approved the final manuscript.

## Supplementary Material

Additional file 1 **Table S1**. Accession numbers for amino acid sequences used for phylogenetic analysis. Click here for file
