# Supplementary material for: Evolution of the CD163 family and its relationship to the bovine gamma delta T cell co-receptor WC1
Source: BMC Evol Biol. 2010 Jun 15;10:181. doi: 10.1186/1471-2148-10-181 (PMC2906472; doi:10.1186/1471-2148-10-181)
Supplement: Additional file 1 — Table S1. Accession numbers for amino acid sequences used for phylogenetic analysis. [file 1471-2148-10-181-S1.DOC]

Additional file 1, Table S1 Accession numbers for amino acid sequences used for phylogenetic analysis.

| Amino acid sequence | Accession (Bovine Genome Database: <http://genomes.arc.georgetown.edu/drupal/bovine/> or GenBank: http://www.ncbi.nlm.nih.gov/) |
| --- | --- |
| BtCD163c-α | GLEAN_14183 |
| BtCD163A | Btgn_tempid_2174, GLEAN_00453 |
| BtWC1 | NP_788824.1 |
| SsWC1 | NP_001116565.1 |
| OaT19 | AAB33544 |
| RnCD163c-α | XP_001059890 |
| PtCD163c-α | XM_521667.2 |
| HsCD163c-α | LOC619207 |
| MmSCART1 | NP_766497.3 |
| MmSCART2 | EF624463.1 |
| ClfCD163c_1 | XP-854338 |
| ClfCD163c_2 | XP-548815 |
| GgCD163_1 | XP-423532.2 |
| GgCD163_2 | XP-001236120.1 |
| GgCD163_3 | XP-001236123.1 |
| GgCD163_4 | XP-416526.2 |
| GgCD163_5 | XP-423001.2 |
| GgCD163_6 | XP-001235946.1 |
| GgCD163_7 | XP-001235941.1 |
| GgCD163_8 | XP-426557.2 |
| GgCD163_9 | XP-001235811.1 |
| GgCD163_10 | XP-001235815.1 |
| GgCD163_11 | XP-001233319.1 |
| GgCD163_12 | XP-424583.2 |
| GgCD163_13 | XP-001236992.1 |
| GgCD163_14 | XP-427519.2 |
| GgCD163_15 | XP-001235597.1 |
| GgCD163_16 | XP-427054.2 |
| GgCD163_17 | XP-428102.2 |
| GgCD163_18 | XP-001233355.1 |
| OraCD163_1 | XP-001518432.1 |
| OraCD163_2 | XP-001518997.1 |
| OraCD163_3 | XP-001517254.1 |
| OraCD163_4 | XP-001510988.1 |
| OraCD163_5 | XP-001520698.1 |
| OraCD163_6 | XP-001518184.1 |
| OraCD163_7 | XP-001519456.1 |
| OraCD163_8 | XP-001513768.1 |
| OraCD163_9 | XP-001506940.1 |
| OraCD163_10 | XP-001509007.1 |
| HsCD163A | Q86VB7 |
| MmCD163A | Q2VLH6 |
| SsCD163A | Q2VL90 |
| HsCD163b | Q9NR16 |
| HsDBMT1 | Q9UGM3 |
| HsCD5 | P06127 |
| HsCD6 | P30203 |
